# Nutritional Status of Children Hospitalized for Parapneumonic Effusion

**DOI:** 10.1371/journal.pone.0094242

**Published:** 2014-04-04

**Authors:** Koen Huysentruyt, Philippe Alliet, Marc Raes, Julie Willekens, Iris De Schutter, Elke De Wachter, Anne Malfroot, Thierry Devreker, Philippe Goyens, Yvan Vandenplas, Jean De Schepper

**Affiliations:** 1 Department of Paediatrics, Universitair Ziekenhuis Brussel, Vrije Universiteit Brussel (VUB), Brussels, Belgium; 2 Department of Paediatrics, Jessa Hospital, Hasselt, Belgium; 3 Department of Paediatric Pulmonology, Cystic Fibrosis Clinic and Paediatric Infectious Diseases, Universitair Ziekenhuis Brussel, Vrije Universiteit Brussel (VUB), Brussels, Belgium; 4 Department of Paediatric Gastro-enterology, Universitair Ziekenhuis Brussel, Vrije Universiteit Brussel (VUB), Brussels, Belgium; 5 Nutrition and Metabolism Unit, Department of Paediatrics, University Children's Hospital Queen Fabiola, Brussels, Belgium; 6 Department of Paediatric Endocrinology, Universitair Ziekenhuis Brussel, Vrije Universiteit Brussel (VUB), Brussels, Belgium; University of Sao Paulo, Brazil

## Abstract

**Background & Aims:**

Among children hospitalized for pneumonia, those with parapneumonic effusion (PPE) are at particular risk for nutritional deterioration. This study aimed to 1) investigate the evolution of the nutritional status during hospitalization and at outpatient follow-up; 2) determine clinical risk factors for weight loss during hospitalization; 3) describe the nutritional interventions for these children.

**Methods:**

Retrospective chart review (January ‘07 - September ‘12) of 56 children with pneumonia, complicated by PPE in two Belgian hospitals for data on body weight and height at admission (t_0_) and discharge (t_1_), and two weeks (t_2_) and one month (t_3_) after discharge. Length of hospitalization (LoS), length of stay in paediatric intensive care (LoS_PICU_) and maximal in-hospital weight loss (t_max_) were calculated and nutritional interventions were recorded.

**Results:**

The median (range) age was 3.5 (1.0–14.8) years. Weight or height was lacking in five (8.9%) children at t_0_ and in 28 (50%) at t_1_; 21.4% was weighed only once during hospitalization. At t_max_, respectively 17/44 and 5/44 children lost ≥5% and ≥10% of their weight. Median (range) LoS and LoS_PICU_ were 18.0 (10–41) and 4.0 (0–23) days. One-fourth received a nutritional intervention. Weight for height at admission (WFH(t_0_)) significantly predicted maximal weight loss (β (95% CI) = −0.34 (−2.0–−0.1); p = 0.03). At t_2_ and t_3_, 13/32 and 5/22 of the children with available follow-up data did not reach WFH(t_0_), whilst in 4/35 and 5/26 body weight remained ≥5% under the weight(t_0_).

**Conclusions:**

One-third of children with pneumonia complicated by PPE and monitored for weight and height, lost ≥5% of their body weight during hospitalization. One-fourth did not reach initial WFH one month after discharge. Those with a higher WFH at admission were at higher risk of weight loss. More attention for monitoring of weight loss and the nutritional policy during and after hospitalization is warranted.

## Introduction

The introduction of the heptavalent pneumococcal conjugate vaccine has decreased the number of hospital admissions for pneumonia [1], [2]. However, recent research demonstrated that acute lower respiratory infections, such as pneumonia, remain a substantial burden on health services worldwide [3]. Parapneumonic effusion (PPE) and pleural empyema has been reported to complicate pneumonia in 3–53% of the cases [4]–[6]. During the last decades, an increase in PPE has been reported [7].

PPE is classically described as a disease spectrum that occurs in three to four stages of increasing complexity, but there are insufficient data to correlate these stages with specific management strategies [8]. There has been extensive research regarding the optimal treatment for childhood pleural empyema, but it still remains a matter of debate [9], [10]. Most studies on treatment strategies focus on immediate outcome; data on longer term consequences of this condition are relatively sparse. It was however recently reported that children frequently missed school in the first month after discharge and that one-fourth of their parents missed work during this period [11].

Also in nutritional research, studies tend to focus on immediate rather than on longer term outcome parameters. Beside the underlying pathology, prolonged fever, chronic pain and reduced intake have been identified as important risk factors for nutritional deterioration of children during their hospital stay [12]. In children hospitalized for PPE, the more severe dyspnoea and increased metabolic requirements, the exudative protein loss and more prolonged periods of fasting for diagnostic and therapeutic procedures are additional clinical risk factors for a more severe nutritional deterioration.

Although in adults malnutrition has been identified as a risk factor for adverse outcome in surgically treated pulmonary disease [13], paediatric research on the course of the nutritional status of children with PPE, on the short as well as the long term, is lacking. Therefore, with this retrospective study we aimed 1) to investigate the change in nutritional status during hospitalization and at outpatient follow-up two weeks and one month after discharge; 2) to determine clinical risk factors for weight loss during hospitalization; 3) to describe the nutritional interventions during hospital stay.

## Methods

The databases of one secondary (Jessa ziekenhuis, Hasselt, Belgium) and one tertiary hospital (UZ Brussel, Brussels, Belgium) were searched for patients that were hospitalized between January 2007 and September 2012 with pneumonia, complicated by PPE. The end date of September 2012 was chosen to avoid duplication of data, since this was the starting date for a prospective study in the tertiary centre. A total of 73 children (twelve in the secondary and 61 in the tertiary hospital) were identified that met the following inclusion criteria: 1) age ≤16 years; 2) confirmed diagnosis of PPE; 3) no underlying disease; 4) no associated disease that markedly affects hydration. The following criteria were set to define a confirmed diagnosis of PPE: acute febrile illness with chest X-ray findings compatible with community acquired pneumonia, in the presence of a significant amount of fluid in the pleural space, defined as ≥10 mm by ultrasound or ≥½ hemithorax on chest X-ray or computerized tomography. Seventeen children from the tertiary hospital were excluded because we did not have sufficient data on the course of their nutritional status during hospitalization or at outpatient follow-up. All of these children were referred by other hospitals directly to the paediatric intensive care unit (PICU) and subsequently transferred back to the referring hospital once the child could be re-admitted to a general paediatric ward.

Medical and nursing files from the remaining 56 children were reviewed for data on the length of hospital stay, days in PICU, nutritional interventions, body weight and height during hospitalization and at outpatient follow-up, two weeks and one month after discharge. Weight data reported maximum two days before discharge were considered as "discharge weight". The “maximal in-hospital weight loss” was defined by subtracting the lowest reported body weight during hospitalization from the weight at admission. If a patient did not lose weight during hospital stay, the weight at admission was used as “minimal weight”. For children that were weighed only once during hospitalization, it was not possible to retrieve a “minimal weight”. Body height, weight for age (WFA) and weight for height (WFH) were converted into z-scores using the Belgian reference data of Roelants et al. [14]. In accordance with internationally accepted definitions, acute under-nutrition was defined as WFH<-2 SD [15]. The study protocol was approved by the ethical committee of UZ Brussel (Commissie Medische Ethiek UZ Brussel). Since only routine data were collected for this research, the need for an informed consent to use the patient files was waived.

Statistics were done using SPSS v 20.0 (SPSS Inc, Chicago Ill., USA) software. Fisher exact or χ^2^- testing was performed for comparison of proportions between groups. The means and medians of continuous variables were compared using a Student's t-test and a Mann-Whitney U test respectively. A paired-samples t-test was used to compare differences in means over time. Regression analysis was used to examine the relationship between the maximal percentage in-hospital weight loss and other variables. A p-value of <0.05 was considered significant. Since there was no significant difference in any of the nutritional outcomes between both hospitals, both patient groups were pooled together for statistical analysis.

## Results

Patient characteristics are shown in **Table 1**. Patient groups from both hospital types were comparable regarding age, sex distribution, length of hospitalization and the number of positive blood cultures. Overall, the median (range) age was 3.5 (1.0–14.8) years old, the median (range) length of hospitalization was 18.0 (10–41) days and the median (range) length of stay in PICU 4.0 (0–23) days. As expected, the number of days in PICU was significantly (p<0.01) higher in the tertiary hospital. In the secondary hospital, Streptococcus pneumoniae was found in all positive blood cultures, whilst in the tertiary hospital this was the case for only four. Of these four children, Streptococcus pneumonia was also isolated from the PPE. In another two patients Staphylococcus aureus was isolated from blood culture, in one of these two children the bacteria was also found in the PPE. Since data on weight and/or height were only reported in 7/13 (53.8%) of the transferred children whose original referral papers could be retrieved, we decided to use the date of admission to the tertiary hospital as a starting point for weight and height in case of referral. The referred patients were all treated with intravenous antibiotics (IV Ab), and this for a median (range) period of 6.0 (1–12) days prior to hospitalization in the tertiary hospital. The children in the secondary hospital were all treated with a combination of IV Ab and a thorax drain, while only five children (11.4%) had this therapy in the tertiary hospital. The other treatments in the tertiary hospital were: IV Ab + drainage and Video-Assisted Thoracoscopic Surgery (VATS) (n = 8); IV Ab + VATS (n = 16); IV Ab alone (n = 15). All children with thorax drainage and/or VATS and 10/15 (66.7%) of the children that were treated with Ab alone, but presenting with loculi in the PPE on ultrasound, were categorized as complicated PPE. The remaining five children were categorized as ‘uncomplicated PPE'. The C-reactive protein (CRP) was higher than 85 mg/l (n = 53) in all children but one, with a median value of 271 mg/l. No significant difference in mean CRP was found between the hospital types (p = 0.08) or between children with a complicated or an uncomplicated PPE (p = 0.88).

**Table 1:** Patient characteristics.

	Tertiary hospital	Secondary hospital	Significancep-value
Male/female (ratio (%))	24/20 (54.5/45.5)	7/5 (58.3/41.7)	0.82
Age* (years)	3. 5 (1.0–14.8)	3.7 (1.0–13.0)	0.44
Hospital stay*(days)	17 (10–37)	19 (13–41)	0.08
PICU stay* (days)	4.5 (0–23)	0.0 (0–4)	<0.01
Transfer from other hospital (%)	15 (34.1)	0 (0)	<0.0001
Positive blood culture (%)	6 (13.6)	3 (25.0)	0.39
Treatment:			<0.0001
- Drain + VATS (%)	8 (12.2)	0 (0)	
- VATS (%)	16 (36.4)	0 (0)	
- Drain (%)	5 (11.4)	12 (100)	
- No drain, no VATS (%)	15 (34.1)	0 (0)	
WFH z score on admission^$^	0.10 (1.3)	−0.19 (1.2)	0.50
Max % in-hospital weight loss*	−3.2 (−14–0)	−4.7 (−13–−1)	0.16
CRP at diagnosis^$^	263.3 (112)	335.6 (146)	0.08

VATS: Video-Assisted Thoracoscopic Surgery; CRP: C-reactive protein; * median and range; ^$^mean (SD).

At admission, weight and height data were present in respectively 56 (100%) and 55 (91.1%) children. Twelve (21.4%) children were weighed only once during hospitalization: one of them was acutely under-nourished (WFH<−2 SD) at admission; a discharge-weight was reported in only 28 (50.0%) children. There was no significant difference between the proportion of children with or without minimal weight reported concerning the following variables: gender (p = 0.67), treatment involving VATS (p = 0.05), positive blood culture (p = 0.26), age (p = 0.73), WFH z-score at admission (p = 0.15), length of hospitalization (p = 0.23) or number of days in PICU (p = 0.05). All children that received a nutritional intervention (14/56) were weighed more than once during their hospital stay, however in only 9/14 children the body weight at the first day of the nutritional intervention was recorded. At outpatient follow-up, both weight and height data were retrieved two weeks and one month after discharge in respectively 35/37 and 26/30 children. Weight data were available at a mean (SD) of 14.92 (5.3) and 35.20 (8.1) days after discharge. Twelve children were assessed at follow-up on both occasions. No significant differences in any of the included variables could be demonstrated between children who were followed-up two weeks after discharge and those who were not, or in those followed-up after one month vs those who were not (data not shown). The number of children with uncomplicated PPE is however too low to allow a separate statistical analysis of their nutritional outcome.

The nutritional status during hospitalization and at outpatient follow-up is displayed in **Table 2**. The mean (SD) WFH z-score at admission was 0.03 (1.3). At admission, 4/56 (7.1%) of the children had a WFA<−2 SD and 2/51 (3.9%) children with both weight and height reported had a WFH<−2 SD. The number of acutely undernourished children increased to 4/43 at the time of maximal percentage weight loss during hospitalization; three of these four children were not undernourished at admission. Of the children who had their weight taken more than once during hospitalization (n = 44), 50 (86.4%) lost weight, with 17 (38.7%) of the children losing ≥5% of their initial body weight and five (11.4%) losing even ≥10%. The mean WFH z-score decreased significantly (p<0.01) during hospitalization: mean (SD) minimal WFH was −0.38 (1.3).

**Table 2:** Nutritional status during hospitalization and at outpatient follow-up.

Parameter	Admission (n = 56)	Minimal weight (n = 44)	2 weeks follow-up (n = 35)	1 month follow-up (n = 26)
WFH z score$	0.03 (1.3)	−0.38 (1.3)*	0.09 (1.1)	0.41 (1.0)*
WFH<−2 SD	2/51 (3.9%)	4/43 (9.3%)	0 (0%)	0 (0%)
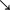 WFH			13/32 (40.6%)	5/22 (22.7%)
≥5% weight loss		17 (38.7%)	4 (11.5%)	5 (19.2%)
≥10% weight loss		5 (11.4%)	1 (2.9%)	1 (3.8%)

$ mean (SD); WFA: weight for age; WFH: weight for height; 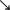
WFH: did not yet reach initial WFH at outpatient follow-up; *Significant (p<0.01) difference compared to mean WFH at admission.

In total, 14/56 (25.0%) of the children received a nutritional intervention during their hospital stay after a median (range) of 6 (2–18) hospital days: 10 children received total parenteral nutrition (TPN), one child received tube feeding and three oral supplements. These interventions were started respectively after a median (range) of 7 (2–13), 8 and 11 (8–18) days. A continuation of oral supplements after discharge was advised in only one child. The median (range) length of hospital stay was significantly (p = 0.03) longer for patients that received TPN (17.5 (10–45) vs 21 (15–35) days), as were the number of PICU days (3 (0–23) vs 10 (0–23); p = 0.01), while the mean WFH on admission was not significantly different (p = 0.41).

**Figure 1:**
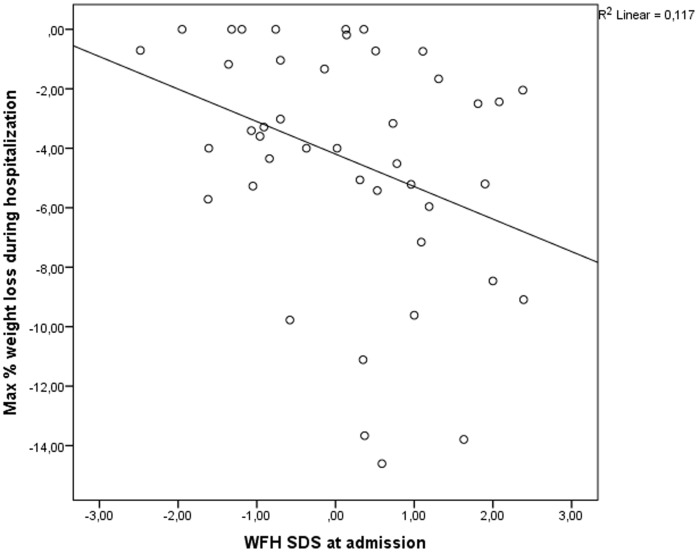
Correlation between max % weight loss during hospitalization and WFH z-score at admission. *WFH: weight for height*.

The relationship between maximal percentage weight loss during hospitalization and the studied variables is presented in **Table 3**. No significant association with age (p = 0.56), gender (p = 0.51), treatment involving VATS (p = 0.66), CRP at diagnosis (p = 0.64), length of hospitalization (p = 0.07) or number of days in PICU (p = 0.72) was found. However, as depicted in **Figure 1**, WFH z-score at admission was a significant predictor for the maximal percentage weight loss during their hospitalization (β (95% CI) = −0.34 (−2.0–−0.1); p = 0.03). When repeating this analysis with only those children that are weighed more than once during hospitalization and a known WHF z score on admission (n = 27), this relationship remained significant (β (95% CI)  = −0.42 (−2.6–−0.1); p = 0.03) with an even higher R^2^ (0.18).

**Table 3:** Predictors of maximal % weight loss during hospitalization.

Variable	R^2^	β (95% CI)	p-value
Age	<0.01	−0.09 (−0.5–0.3)	0.56
Gender	0.01	−0.10 (−3.3–1.6)	0.51
Treatment (involving VATS)	<0.01	−0.07 (−3.0–1.9)	0.66
WFH z-score at admission	0.12	−0.34 (−2.0–−0.1)	0.03
Length of hospital stay	0.08	−0.28 (−0.4–0.0)	0.07
Length of PICU stay	<0.01	−0.06 (−0.3–0.2)	0.42

Univariate linear regression analysis of factors influencing maximal % weight loss during hospitalization.

At outpatient follow-up two weeks and one month after discharge respectively 19/35 (54.3%) and 13/26 (50.0%) children had not reached the body weight reported at admission and 4/35 (11.5%) and 5/26 (19.2%) had a body weight that was ≥5% under the weight at admission. When also taking linear growth into account, in 13/32 (40.6%) of the children at two weeks follow-up and in 5/22 (22.7%) at one month follow-up the initial WFH z-score was still not reached.

## Discussion

To the best of our knowledge, this is the first study on the nutritional consequences of PPE in hospitalized children on short and longer term. We found that of the children who were weighed more than once during hospitalization, 86% lost weight during their hospital stay, with 45% and 13% of these children losing respectively ≥5% and ≥10% of their body weight. The maximal percentage weight loss during hospitalization was significantly predicted by the WFH z-score on admission. Nonetheless, despite a median length of hospital stay of 18 days, one-fifth was weighed only once during hospitalization and in only one quarter of the children a nutritional intervention was set-up. Our data also demonstrate the nutritional impact of this acute-onset infectious disease on longer term: nearly one-fourth of the children that were followed-up one month after discharge did not yet reach the WFH z-score at admission.

Our finding of 3.9% acute under-nutrition at admission is somewhat lower than previously reported in Belgian hospitals [16]. This might be explained by the fact that we excluded all children with an underlying chronic condition from our study population, as these children were shown to be more prone to acute under-nutrition at admission [16]. A Turkish study reported that 65% of their children with PPE and empyema had a WFA <90% of the reference standard [17] and in a Brazilian tertiary hospital, a prevalence of 20% acute under-nutrition was reported [18]. The better socio-economic status in Western Europe, as well as the use of a WFA cut-off of <−2 SD can explain the lower prevalence in our study.

Overall, the literature on the evolution of the nutritional status of children during hospitalization is limited [19] and we are unaware of any reports focusing on children with PPE. We recently reported that 40% of children hospitalized in Belgium were losing ≥5% of their body weight [16], taking discharge weight and not minimal weight into account. In a less recent French study of Sermet-Gaudelus et al., it was reported that one-fourth of the children hospitalized for various medical conditions lost >5% of their body weight during hospitalization [12]. Interestingly, Rocha et al described that three quarter of the children hospitalized with pneumonia lost weight, despite having an adequate nutritional status at diagnosis [20]. Our data also showed that children with a higher WFH z-score on admission had a greater percentage weight loss during hospitalization. We hypothesize that there is less attention for a proper nutritional policy in children that seem to have a better nutritional status at admission, and that reduced intake in these children is overlooked more easily. On the other hand, the WFH z score at admission gives only a limited explanation (12%) for the in-hospital weight loss. Since PPE is usually a secondary progression of a preceding pneumonia, it is possible that lengthy illness prior to admission may influence correlation of WFH with in-hospital weight loss. These results demonstrate that nutritional screening, which encompasses not only a description of the current nutritional status but also the presence of other risk factors such as reduced intake or pain, is an important part of a nutritional care program.

More frequent weight monitoring was observed in those children in whom a nutritional intervention was set-up. In only 25% of the PPE children a nutritional intervention was set-up, mostly after the first week of hospitalization. We suspect that the majority of the patients in our cohort would have benefit from a hyper-caloric and high protein nutritional regime, at least during convalescence, since the presence of inflammation and reduced intake lead to a catabolic state, characterized by the presence of catabolic hormones, inflammatory cytokines and an acute phase protein response. This will lead to a net loss of protein and fat mass, which is further aggravated by protein loss via pleural exudate. On the other hand, overfeeding due to parenteral feeding should be avoided in children with PPE, to reduce the risk of hypercapnia and fluid overload. We also found that patients who received TPN had a significantly longer hospital stay and a significantly longer stay on PICU. It is plausible that TPN was started in these patients because they were expected to be hospitalized for a longer period and were unable to ingest adequate amounts of food. On the other hand, it may also be true that the longer hospital stay was caused by an interference of TPN with rate of bacterial clearance. Shouman et al demonstrated recently that reducing the amount of lipids in TPN was associated with a more rapid sterilization of blood cultures in preterm neonates [21]. However, the design of our study does not allow us to determine any causal relationships.

Another important finding in our study was that nearly one-fourth of the children that was followed-up one month after discharge did not reach their initial WFH z-score, which was possibly even lower than their pre-disease WFH z-score. In only one patient oral supplements were continued after discharge, while no records of post-discharge dietetic advice could be found for any of the other patients. We suspect that additional energy during the recovery phase at home could improve general well-being with a faster resumption of normal sporting and scholar activities. Prospective interventional studies assessing both physical performance and quality of life at longer term are needed to support this hypothesis.

Despite the fact that at least half of the children were hospitalized for two weeks, one-fifth of our population was weighed only once during hospitalization. This finding is not new: the nutrition audit by Hartman [22] in Israel showed that 24% of the children had no weight recorded during hospitalization and the Australian report of O'Connor et al [23] described that only 27% of the patients had both their weight and height recorded. Interestingly, we were surprised to find data on weight and/or height in only 54% of the transferred children, although this figure is still higher than the 8% found in a Dutch study on communication between general practitioners and specialists concerning adults with head and neck cancer [24].

Due to the retrospective design of our study, we are lacking anthropometric data of a part of our study population at each study moment. It is possible that the patients described at outpatient follow-up are a selection of the most at-risk patients. On the other hand, it is also plausible that patients that didn't report at outpatient follow-up are those patients with the lowest socio-economic status, and thus more at risk for poor nutritional recovery at home. Nonetheless, we feel confident that our conclusions are applicable to the entire study cohort, since we could not demonstrate any differences in any of the studied parameters between children seen at follow-up visits and those who are not. We are also well aware that we have included patients at both ends of the PPE disease spectrum. However, the finding of high CRP-levels at diagnosis, which have been shown to be useful in distinguishing viral from bacterial pneumonia [25] and in predicting the risk for developing complicated PPE's [26], in our population strengths our believe that all children were suffering from a more severe form of PPE, even the eight percent children who were categorized as an uncomplicated form. A direct comparison of our study cohort with children hospitalized for acute pneumonia was not possible, as these children are generally hospitalized during a period of only three to four days, and no routine follow-up at the hospital is scheduled for these children afterwards. We would however recommend this for future research as it may be helpful in assessing the impact of severity of disease itself on the nutritional state.

In conclusion, one-third of previously healthy children with community acquired pneumonia and PPE and monitored weight lost ≥5% of their body weight during hospitalization. Children with a higher WFH z-score at admission were more at risk for greater weight loss during hospitalization, which emphasizes the need for nutritional screening and follow-up during hospitalization as an important part of a nutritional care program. One-fourth of the children reported at outpatient follow-up after one month, did not reach the WFH z-score described at admission. Prospective nutritional interventional studies are needed to confirm these findings and to assess the effect on both physical performance and quality of life at longer term.
